# The Humoral Immune Response to BNT162b2 Vaccine Is Associated With Circulating CD19+ B Lymphocytes and the Naïve CD45RA to Memory CD45RO CD4+ T Helper Cells Ratio in Hemodialysis Patients and Kidney Transplant Recipients

**DOI:** 10.3389/fimmu.2021.760249

**Published:** 2021-12-03

**Authors:** Anila Duni, Georgios S. Markopoulos, Ioannis Mallioras, Haralampos Pappas, Efthymios Pappas, Vasileios Koutlas, Eirini Tzalavra, Gerasimos Baxevanos, Silvia Priska, Konstantina Gartzonika, Michael Mitsis, Evangelia Dounousi

**Affiliations:** ^1^ Department of Nephrology, University Hospital of Ioannina, Ioannina, Greece; ^2^ Department of Surgery and Kidney Transplant Unit, University Hospital of Ioannina, Ioannina, Greece; ^3^ Laboratory of Hematology - Unit of Molecular Biology, University Hospital of Ioannina, Ioannina, Greece; ^4^ Renal Unit, General Hospital of Filiates, Filiates, Greece; ^5^ Internal Medicine Department, Hatzikosta General Hospital of Ioannina, Ioannina, Greece; ^6^ Department of Nephrology, School of Medicine, University of Ioannina, Ioannina, Greece; ^7^ Microbiology Laboratory, Faculty of Medicine, School of Health Sciences, University of Ioannina, Ioannina, Greece

**Keywords:** SARS-COV-2 vaccination, hemodialysis, kidney transplant recipients, anti-SARS-CoV2 antibodies, CD19+ B lymphocytes, naïve CD4+CD45RA+ T helper cells, memory CD4+CD45RO+ T helper cells

## Abstract

**Background:**

The humoral and cellular immune responses to SARS-COV-2 vaccination remain to be elucidated in hemodialysis (HD) patients and kidney transplant recipients (KTRs), considering their baseline immunosuppressed status. The aim of our study was to assess the associations of vaccine-induced antibody responses with circulating lymphocytes sub-populations and their respective patterns of alterations in maintenance HD patients and KTRs.

**Materials and Methods:**

We included 34 HD patients and 54 KTRs who received two doses of the mRNA-vaccine BNT162b2. Lymphocyte subpopulations were analyzed by flow cytometry before vaccination (T0), before the second vaccine dose (T1) and 2 weeks after the second dose (T2). The anti-SARS-CoV2 antibody response was assessed at T1 and at T2.

**Results:**

31 HD patients (91.8%) and 16 KTRs (29.6%) became seropositive at T2. HD patients who became seropositive following the first dose displayed higher CD19+ B lymphocytes compared to their seronegative HD counterparts. A positive correlation was established between CD19+ B cells counts and antibody titers at all time-points in both groups (p < 0.001). KTRs showed higher naïve CD4+CD45RA+ T helper cells compared to HD patients at baseline and T2 whereas HD patients displayed higher memory CD45RO+ T cells compared to KTRs at T2. The naïve CD4+CD45RA to memory CD4+CD45RO+ T helper cells fraction was negatively associated with antibody production in both groups.

**Conclusions:**

Our study provides a potential conceptual framework for monitoring vaccination efficacy in HD patients and KTRs considering the correlation established between CD19+ B cells, generation of memory CD4+ T helper cells and anti SARS-CoV2 antibody response to vaccination.

## Introduction

The COVID-19 pandemic poses unique challenges to patients undergoing maintenance renal replacement therapy and kidney transplant recipients (KTRs) with available evidence until now indicating a higher morbidity and mortality trend following infection compared with the general population (1, 2). Despite increased rates of vaccination among these vulnerable populations, the adequacy of the respective generated immune responses remains a subject of concern and ongoing evaluation. The complex derangement of the immune system as occurs both in end-stage kidney disease (ESKD) and kidney transplantation has been directly associated with an increased susceptibility to infections and impaired response to vaccination in these patients (3, 4). The uremic milieu of ESKD and the immunosuppressive and immunomodulatory medications administered in the setting of kidney transplantation affect directly both the humoral and cell-mediated immunity (5–8). Overall, decreased numbers of circulating T, B and NK lymphocytes as well as altered CD4+ and CD8+ T cell responses and low antibody production by B lymphocytes following stimulation have been found in hemodialysis patients (5, 6). Likewise, altered T-cell activation, proliferation, cytokine production and cytotoxicity and B‐cell lymphopenia represent the hallmark of the immunosuppressed state of kidney transplantation (7, 8).

Available reports regarding the humoral response to COVID-19 vaccination in patients receiving maintenance hemodialysis, show better results compared to the poor antibody response of KTRs (9). Yet lower overall antibody titers in maintenance hemodialysis patients as compared to individuals without kidney disease have been reported (10–12).

Specific T-cell memory responses elicited by the SARS-CoV-2 infection might play a significant protective role even in the absence of specific antibodies (13, 14). Despite an abundance of data regarding generation of anti-S protein IgG and virus-specific neutralizing antibody responses, T cell responses following vaccination, including patterns of naive T cell activation and differentiation into effector cells have not been fully evaluated. Reduced numbers of NK cells in peripheral blood together with NK cell hyperactivation and dysfunction have been found in patients with severe Covid-19 disease, whereas there is scarce and controversial evidence regarding NKT cells responses in this setting (15, 16). Furthermore models of vaccine-dependent generation of antigen-specific memory NK cells and the utilization of NKT cell agonists as novel immune adjuvants in the setting of vaccination have received increasing attention recently (17, 18). With regard to the baseline immunosuppressed state associated with ESKD and transplantation, there is a paucity of data regarding the analysis of peripheral blood lymphocyte sub-populations, their patterns of change following vaccination against SARS COV-2 as well as their respective immunologic and clinical significance in such context.

Considering that the immune response is orchestrated by the specialized subpopulations of lymphocytes, the aim of our study was to evaluate and compare the antibody response status together with vaccine-induced alterations in circulating lymphocytes subsets, including B cells, CD4+ and CD8+ T cells, naïve and memory T lymphocytes subpopulations, as well as well as NK and NKT cells, following the administration of two doses of the BNT162b2 vaccine in a cohort of maintenance hemodialysis (HD) patients and KTRs.

## Materials and Methods

This prospective study was conducted in the Hemodialysis Unit of the Nephrology Department and the Kidney Transplant Unit of the University Hospital of Ioannina. The study protocol was approved by the Ethical Committee of our hospital (8/14-4-2021) and has been registered on ClinicalTrials.gov (NCT04932876). We included in our study 34 chronic HD patients and 54 KTRs with no previous history of SARS-CoV2 infection, who received two doses of the mRNA-vaccine BNT162b2. The two doses of the BNT162b2 vaccine were administered intramuscularly and 21 days apart. All patients provided signed informed consent for participation in the study. Exclusion criteria included presence of active autoimmune disease, active malignancy and chronic infections (HBV, HCV, HIV). In addition, patients with acute infections, recent surgical procedures within the last 2 weeks from vaccination or organ transplantation within the last six months from vaccination were excluded from the study.

Clinical data, including the maintenance immunosuppressant regimen were recorded from the patients’ medical files. Furthermore, baseline routine laboratory tests and blood levels of immunosuppressive medications (tacrolimus and cyclosporine) were obtained at all time points. In KTRs estimated glomerular filtration rate (eGFR) was calculated using the CKD-EPI formula and 24-hours protein urine excretion was assessed.

### Anti-SARS-CoV2 Antibody Response

Serologic response was assessed by using the ARCHITECT IgG II Quant test (Abbott). Titers >50 arbitrary units (AU)/ml were considered positive for seroconversion (detection range, 6.8–80,000 AU/ml); positive agreement, 99.4%; negative agreement, 99.6%. The anti-SARS-CoV2 antibody response against the spike protein was assessed at two time points, immediately before the second vaccine dose (T1) and 2 weeks after administration of the second dose (T2).

### Flow Cytometry Analysis

**Figure 1:**
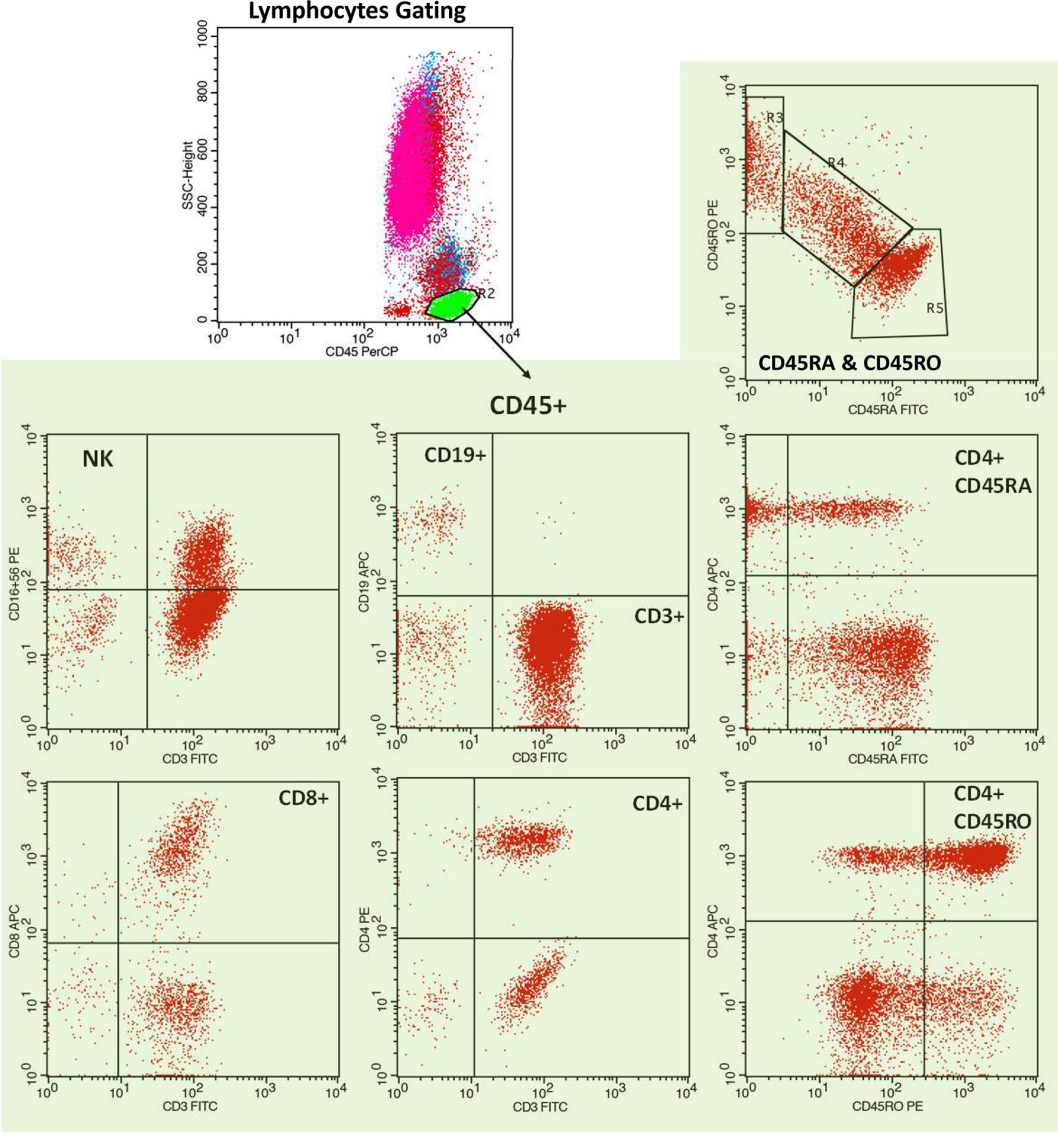
Representative flow cytometric analysis of a hemodialysis patient. Representative dot plots depicting lymphocyte gating with B-lymphocytes (CD19+), and T lymphocytes (CD3+), CD4+ T cells, CD8+ T cells, naïve (CD45RA+) and memory (CD45RO+) T cell isoforms, naïve (CD4+CD45RA) T helper cells and memory (CD4+CD45RO) T helper cells, NK cells (CD3-CD16+CD56+) and NKT cells (CD3+CD16+CD56+).

Conjugated monoclonal antibodies were used for four-color flow cytometric analysis performed in a FACScalibur cytometer (Becton Dickinson). The particular anti-human antibodies used were: CD3-FITC (Clone UCHT1), CD4-PE (Clone MEM-241), CD4-APC (Clone MEM-241), CD8-APC (Clone MEM-31), CD16-PE (Clone MEM-154), CD19-APC (Clone LT19), CD45-PerCP (Clone MEM-28), CD45RA-FITC (Clone MEM-56), CD45RO-PE (Clone UCHL1) and CD56-PE (Clone LT56), purchased from EXBIO, Praha SA. 100 μl of whole-blood was added to flow cytometry (FC) tubes and incubated with respective antibodies according to manufacturer’s instructions. 500 μl of Versalyse (Beckman Coulter) was added and incubated for 10 minutes at room temperature (18-25°C) protected from light, to lyse red blood cells. Samples were processed immediately for four-color FC analysis. The data were analyzed using the CellQuest V3.1 software (Becton Dickinson). Lymphocyte subpopulations, including NKT cells and NK cells, CD19+ B lymphocytes, CD45RA+ (naïve) CD45RA+CD45RO+ (transient) and CD45RO+ (memory) T cell isoforms, CD4+ T helper cells, CD8+ T cells, CD4+CD45RA (naive) T helper cells, CD4+CD45RO (memory) T helper cells and their ratio were analyzed by FC within 24 hours from sampling at three time points, at baseline, i.e. before vaccination (T0), immediately before the second vaccine dose (T1), and 2 weeks following administration of the second vaccine dose (T2) (**Figure 1**). In specific and as previously described, whole blood from each individual was analyzed by flow cytometry, for the presence of specific lymphocyte subpopulations at T1 and T2, at the same time points that antibody response was evaluated.

### Statistical Analysis

Means and standard deviations were used to express all outcome measures while the median and IQR were reported in cases where normality was not met, under the Shapiro Wilk criterion. A mixed models approach was adopted to examine differences within the levels of each outcome across the three time points but also between KTRs and HD patients. Differences between responders and non responders were also examined where the sample size was not too small. The Tukey’s HSD criterion was applied to adjust for the significance level after multiple comparisons. Correlations between different parameters were established using Spearman’s Rho criterion. Depiction of correlations was performed using Regression Variable Plots. The SPSS v23.0 software was applied to analyze all data and the significance level was set at 0.05 in all cases.

## Results

The main demographics, clinical and laboratory parameters of the HD patients and KTRs are depicted in **Table 1**. Hemodialysis patients were significantly older than KTRs (69.4 ± 12.3 vs 58.2 ± 9.7, p<0.001) and had significantly lower levels of hemoglobin in comparison to KTRs (10.8 ± 1.1 vs 13.5 ± 1.9 g/dl, p<0.001).

**Table 1:** Demographics, clinical and laboratory parameters in hemodialysis patients and kidney transplant recipients.

	Hemodialysis patients (n = 34)	Kidney Transplant Recipients (n = 54)	p
Age, yr	69.4 ± 12.3	58.2 ± 9.7	<0.001
Male gender	23, 67.6%	38, 70.4%	0.35
BMI, kg/m^2^	25.9 ± 4.0	25.1 ± 4.1	0.35
Diabetes mellitus	9, 26.5%	10, 18.5%	0.38
History of Cancer	1, 5.6%	5, 9.3%	0.62
Time on dialysis, years	12.39 ± 8.36	5.71 ± 4.68	<0.001
Kt/V	1.6 ± 0.3		
Time from Kidney Transplant, years		11.9 ± 8.3	
Donor type (deceased, live, both)		35, 64.8%/18, 33.3%/1, 1.9%	
ABO group			
O	11, 32.4%	22, 42.3%	0.44
A	12, 35.3%	21, 40.4%	
B	9, 26.5%	7, 13.5%	
AB	2, 5.9%	2, 3.8%	
Hb, g/dl	10.8 ± 1.1	13.5 ± 1.9	<0.001
WBC, 10^3^/ml	8184 ± 2667	8165 ± 9648	0.99
Induction, Anti-CD25		54, 100%	
CNI		54, 100%	
Tacrolimus		41, 75.9%	
Cyclosporine		13, 24.1%	
MMF/MPA		50, 92.6%	
Steroids		46, 85.2%	
Tacrolimus+MMF/MPA		39, 72.2%	
Tacrolimus+MMF/MPA+Steroids		35, 64.7%	
Tacrolimus levels, ng/ml		6.6 ± 1.4	
Cyclosporine T0 levels, ng/ml		103.7 ± 22.8	
eGFR, ml/min per 1.73m^2^		52.7 ± 17.5	
Urine Protein, mg/24h		329.6 ± 411.3	

BMI, body mass index; CNI, calcineurin inhibitor; eGFR, estimated glomerular filtration rate; MMF, mycophenolate mofetil; MPA, mycophenolic acid.

### Overview of the Humoral Immune Response Following Administration of BNT162b2 Vaccine

**Figure 2:**
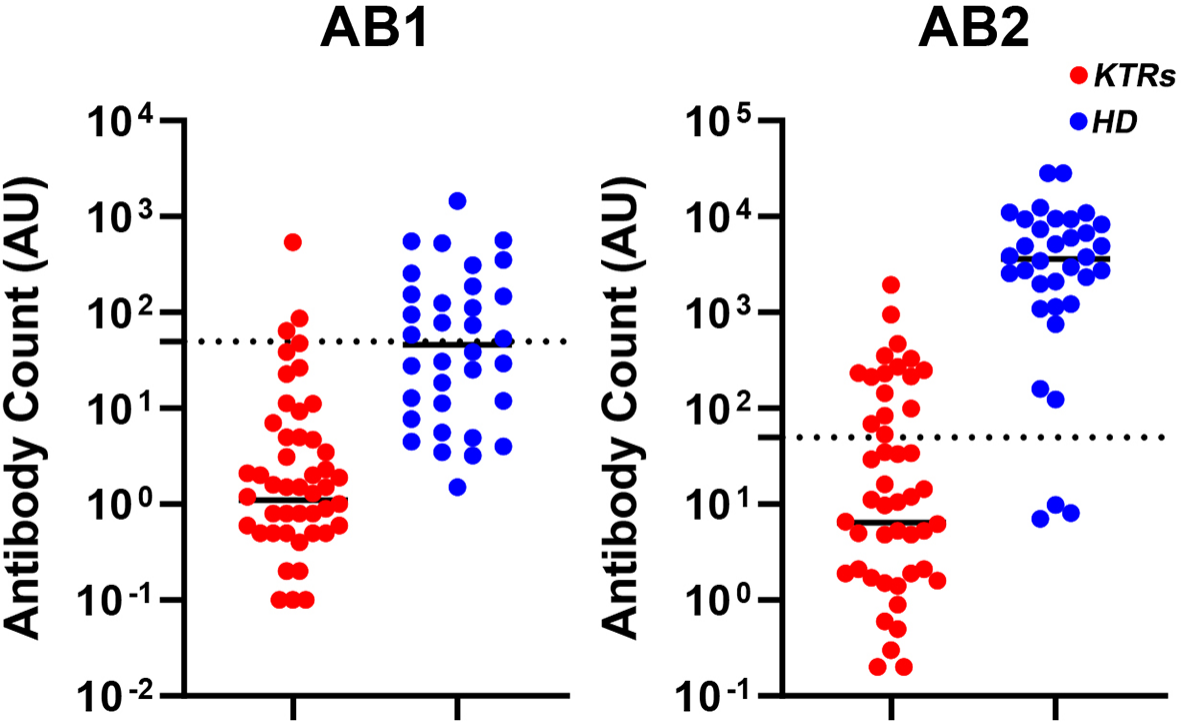
Scatter plots representing individual values for each patient of the anti-SARS-CoV2 antibody titers against the spike protein immediately before the second vaccine dose (T1) and 2 weeks after administration of the second dose (T2) in hemodialysis patients and kidney transplant recipients. AB1, antibody response at T1; AB2, antibody response at T2; HD, hemodialysis patients; KTRs, kidney transplant recipients.

With regard to antibody response status in patients undergoing maintenance dialysis, 17 (50%) of them became seropositive following administration of the first vaccine dose (T1), which increased to overall 31 patients (91.8%) becoming seropositive two weeks after administration of the second vaccine dose (T2). On the other hand, only 3 (5.6%) patients achieved seropositivity in the KTRs group after administration of the first vaccine dose (T1) and subsequently 16 KTRs (29.6%) following the second vaccine dose (T2) (**Figure 2**). In the same line, the mean level of antibody titers was significantly higher in HD patients in comparison to KTRs in both measurements, following the first and second vaccine doses respectively (156.9 ± 279.8 vs 16.9 ± 74.6 g/dl, p <0.001 and 5759.9 ± 6771.6 vs 113.9 ± 300.0 g/dl, p < 0.001).

**Table 2** presents the main demographics, clinical and laboratory parameters of the HD patients and KTRs with regard to their antibody response status and comparisons within each group between patients who converted and those who did not convert following the second vaccine dose. In specific, among KTRs, non-responders displayed lower eGFR levels (48.9 ± 17.2 vs 61.6 ± 15.4 ml/min per 1.73m^2^, p = 0.014) and their regimen included more immunosuppressive medications (Tacrolimus+MMF/MPA+ Steroids 28, 73.7% vs 7, 43.8%, p = 0.035) as compared to responders.

**Table 2:** Demographics, clinical and laboratory parameters in hemodialysis patients and kidney transplant recipients presented as responders versus non responders in each group after the second dose of vaccine.

	Hemodialysis patients (n = 34)		p	Kidney Transplant Recipients (n = 54)		p
	Responders (n= 31)	Non-Responders (n=3)		Responders (n=16)	Non-Responders (n=38)	
Age, yr	69.2 ± 12	71.3 ± 17.6	0.78	55.01 ± 2.23	59.5 ± 7.48	0.18
Male sex	22, 71.0%	1, 33.3%	0.24	13, 81.3%	25, 65.8%	0.26
BMI, kg/m^2^	25.23 ± 4.19	23.77 ± 2.86	0.56	25.85 ± 3.99	25.98 ± 4.05	0.92
Diabetes mellitus	7, 22.6%	2, 66.7%	0.16	3, 18.7%	7, 18.4%	0.98
History of Cancer	1, 6.7%	–		1, 6.2%	4, 10.5%	0.62
Time on dialysis, years	5.81 ± 4.88	4.66 ± 1.53	0.69			
Time from Transplant, years				12.94 ± 9.62	11.45 ± 7.70	0.55
Donor type						
Deceased				9, 56.2%	26, 68.4%	0.55
Live				7, 43.8%	11, 28.9%	
Both				–	1, 2.6%	
ABO group			0.57			0.27
A	10, 32.2%	2, 66.7%		4, 25.0%	17, 47.2%	
AB	2, 6.5%	–		–	2, 5.6%	
B	8, 25.8%	1, 33.3%		2, 12.5%	5, 13.9%	
O	11, 35.5%	–		10, 62.5%	12, 33.3%	
Hb, g/dl	10.97 ± 1.00	9.37 ± 1.01	0.013	14.11 ± 1.85	13.30 ± 1.88	0.15
WBC, 10^3^/ml	8326 ± 10050	6496 ± 4010	0.76	7961 ± 2751	8277 ± 2663	0.69
Tacrolimus				10, 62.5%	31, 81.6%	0.13
Cyclosporine				6, 37.5%	7, 18.4%	0.13
MMF/MPA				12, 75.0%	38, 100.0%	0.006
Steroids				12, 75.0%	34, 89.5%	0.17
Tacrolimus+MMF/MPA				8, 50.0%	31, 81.6%	0.018
Tacrolimus+MMF/MPA+Steroids				7, 43.8%	28, 73.7%	0.035
Tacrolimus levels, ng/ml				6.2 ± 1.7	6.8 ± 1.2	0.24
Cyclosporine T0 levels, ng/ml				102.6 ± 19.2	104.5 ± 26.0	0.88
eGFR, ml/min per 1.73m^2^				61.6 ± 15.4	48.9 ± 17.2	0.014
Urine Protein, mg/24h				307 ± 550	339 ± 345	0.80

BMI, body mass index; CNI, calcineurin inhibitor; eGFR, estimated glomerular filtration rate; MMF, mycophenolate mofetil; MPA, mycophenolic acid.

### Distribution of Specific Immune Cell Subsets Before and After Vaccination Against SARS-CoV-2

**Figure 3:**
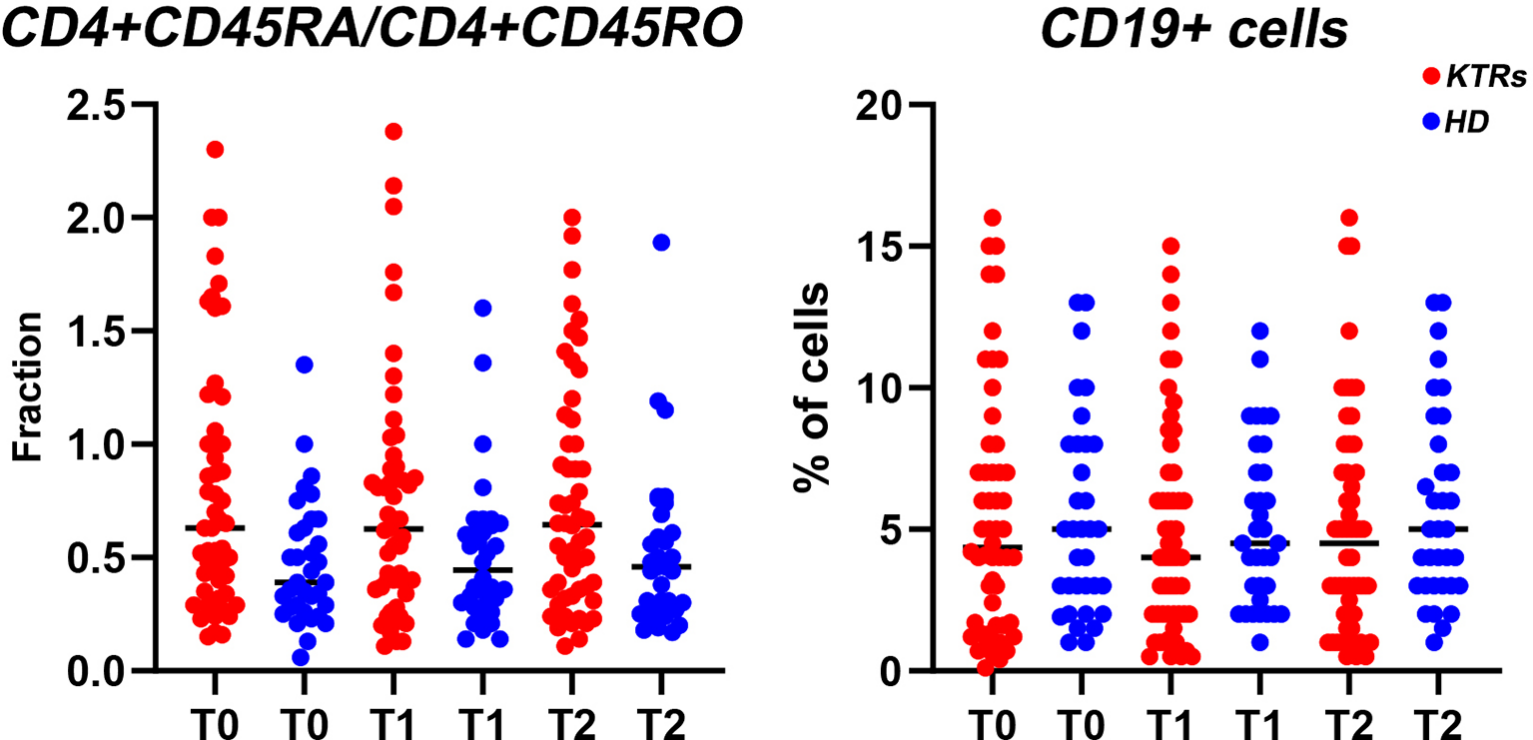
Scatter plots representing individual values for each patient of the fraction of CD4+CD45RA/CD4+CD45RO T-helper cells and the percentage of CD19+ B cells respectively at baseline (T0), immediately before the second vaccine dose (T1) and 2 weeks after administration of the second dose (T2) in hemodialysis patients and kidney transplant recipients. HD, hemodialysis patients; KTRs, kidney transplant recipients.

CD19+ B lymphocytes produce antibodies and are in control of the humoral immune response. CD19+ B lymphocyte counts (normal reference values 6-22%) before vaccination were 5.35% and 5.45% of total lymphocytes in the HD patients and KTRs, respectively. No significant differences were found between the two patients groups regarding CD19+ B cell counts at any time points with p-values exceeding 0.96 in all cases. Yet, HD patients who developed an antibody response with IgG antibodies against the spike receptor-binding domain (RBD) of SARS-CoV-2 above 50 AU/ml following administration of the first vaccine dose, had at all times higher levels of CD19+ B cell counts in comparison to HD patients who failed to generate an antibody response at this time point. Accordingly, the mean differences for CD19+ B cell counts at T0, T1 and T2 respectively were (3,118 ± 1.759% vs 7.588±,3.355% p=0.015), (3.117 ± 1.484% vs 7.118 ± 2.601%, p=0.049), (3.323 ± 1.446% vs 8.147 ± 3.081%, p=0.006) (**Figure 3**). However, this difference was not maintained with regard to antibody response following the second vaccine dose, neither did we detect any differences between CD19+ B lymphocytes and antibody response status in KTRs. Yet, one should take into consideration the fact that nearly half of our HD cohort already had a positive serology following the first vaccine dose as compared to only 4 (7%) of KTRs.

We further distinguished the T cell compartment in naïve T cells (CD45RA+) which are unprimed lymphocytes and memory T cells (CD45RO+) which have encountered antigen and respond faster and with increased intensity on antigenic stimulation compared with (CD45RA+) naïve T cells (19). Analysis of the CD45RA+ (naïve) CD45RA+CD45RO+ (transient) and CD45RO+ (memory) isoforms as expressed by T cell subsets, revealed no differences with regard to either CD45RA+ or CD45RA+CD45RO+ levels between KTRs and HD patients at any time point, neither within each sub-group of responders and non-responders. On the other hand, hemodialysis patients displayed higher mean levels of the CD45RO+ T cells compared to KTRs at T2 (39.706 ± 8.792% vs 33.185 ± 9.481%, p=0.020) independently of the antibody response status.

CD4+ T helper cells are regarded as the orchestrators of cellular immunity, with several roles in antiviral responses, including the assistance to B cell activation, generation of antibody-producing plasmocytes and memory B cells, the expression of cytokines as well as the generation of cytotoxic and memory CD8+ T cell subpopulations (20). CD4+ T helper cells significantly increased in KTRs at T2 as compared to baseline (54.185 ± 11.63% vs 49.389 ± 10.967%, p=0.004). With regard to HD patients, a declining trend of CD4+ T cells from T0 to T1 (44.000 ± 9.032% vs 42.853 ± 7.207%) was observed which was subsequently followed by a significant increase of CD4+ T cell counts at T2 as compared to T1 (48.294 ± 11.559% vs 42.853 ± 7.207%, p=0.043). Overall, KTRs showed higher CD4+ T cell counts in comparison to the HD patient group at the T2 time point.

The central role of CD4+ cells in immunity led us to further study the transition from CD4+CD45RA (naive) to CD4+CD45RO (memory) T helper cells during the vaccination period and in conjunction to the antibody response. Accordingly, KTRs showed higher CD4+ CD45RA+ T helper cell counts in comparison to HD patients at T0 (18.852 ± 9.252% vs 12.618 ± 6.642%, p=0.015) as well as at T2 (19.167 ± 10.136% vs 13.824 ± 7.129%, p=0.057) though marginally significant at this time point, independently of the antibody response status. No differences were found between KTRs and HD patients at any time point, nor between the responders and non-responders within each group with regard to CD4+CD45RO T helper cells.

CD8+ T cells showed a decreasing trend in both the HD and KTRs patient groups at T2 as compared to baseline. Thus, CD8+ T cells counts at T2 as compared to T0 respectively were 24.296 ± 11.409% vs 30.167 ± 11.119% (p <0.001) in KTRs and 23.206 ± 9.984% vs 28.118 ± 9.546% (p=0.008) in HD patients. Furthermore, the CD4+/CD8+ ratio significantly increased at T2 compared to baseline in both HD (2.722 ± 2.051 vs 1.885 ± 1.085, p=0.029) and KTRs (2.941 ± 2.017 vs 1.929 ± 0.986, p<0.001).

Natural killer cells and NKT cells, are considered to bridge the innate and acquired arms of the immune system, thus organizing adaptive immune responses and immunoregulation (21). Regarding NKT-cells (CD3+CD16+CD56+) counts, an increase was observed in KTRs at T1 (5.967 ± 6.705) and T2 (7.520 ± 6.273%) as compared to baseline (3.978 ± 4.377%). Hemodialysis patients displayed a different pattern of change in NKT cell counts. NKT cells levels increased substantially from T0 to T1 (2.465 ± 1.583% vs 6.180 ± 7.186% p=0,014), whereas a decrease was subsequently observed at T2 (2.941 ± 2.373%), with their levels albeit remaining higher than baseline. In addition, KTRs displayed higher levels of NKT cells at T2 compared to their hemodialysis counterparts (7.520 ± 6.2735 vs 2.941 ± 2.373%, p=0.001). NK cells (CD3-/CD16+/CD56+) remained stable at all time points within each sub-group, with HD patients in general displaying higher NK cell counts compared to KTRs.

### The Antibody Response Status Is Associated With the CD19+ Lymphocyte Subpopulation and the Fraction of CD45RA Naïve to CD45RO Memory CD4+ T Helper Cells

To gain further insight into the cellular immune responses that lead to COVID-19 antibody production, we examined whether antibody levels were associated with CD19+ B lymphocyte counts. Accordingly, a positive correlation was established between CD19+ B cells counts in the circulation and the IgG antibody levels at all time-points (p <0.001) (**Supplementary Table 1**). Collectively, our data support that even though CD19+ B cell counts are below the normal reference values in HD patients and KTRs, their positive correlation to antibody production supports the induction of the humoral immune response following BNT162b2 vaccination.

Furthermore, in order to study the transition between naïve and memory CD4+ T helper cells we analyzed the fraction of CD4+CD45RA/CD4+CD45RO cells, with a lower fraction signifying a larger percentage of memory cells compared to naïve cells (**Figure 3**). We found that the CD4+CD45RA/CD4+CD45RO fraction was negatively associated to antibody production, in both time-points (**Supplementary Table 2**), thus allowing us to conclude that the production of memory T helper cells is directly associated with the antibody response status.

**Figure 4:**
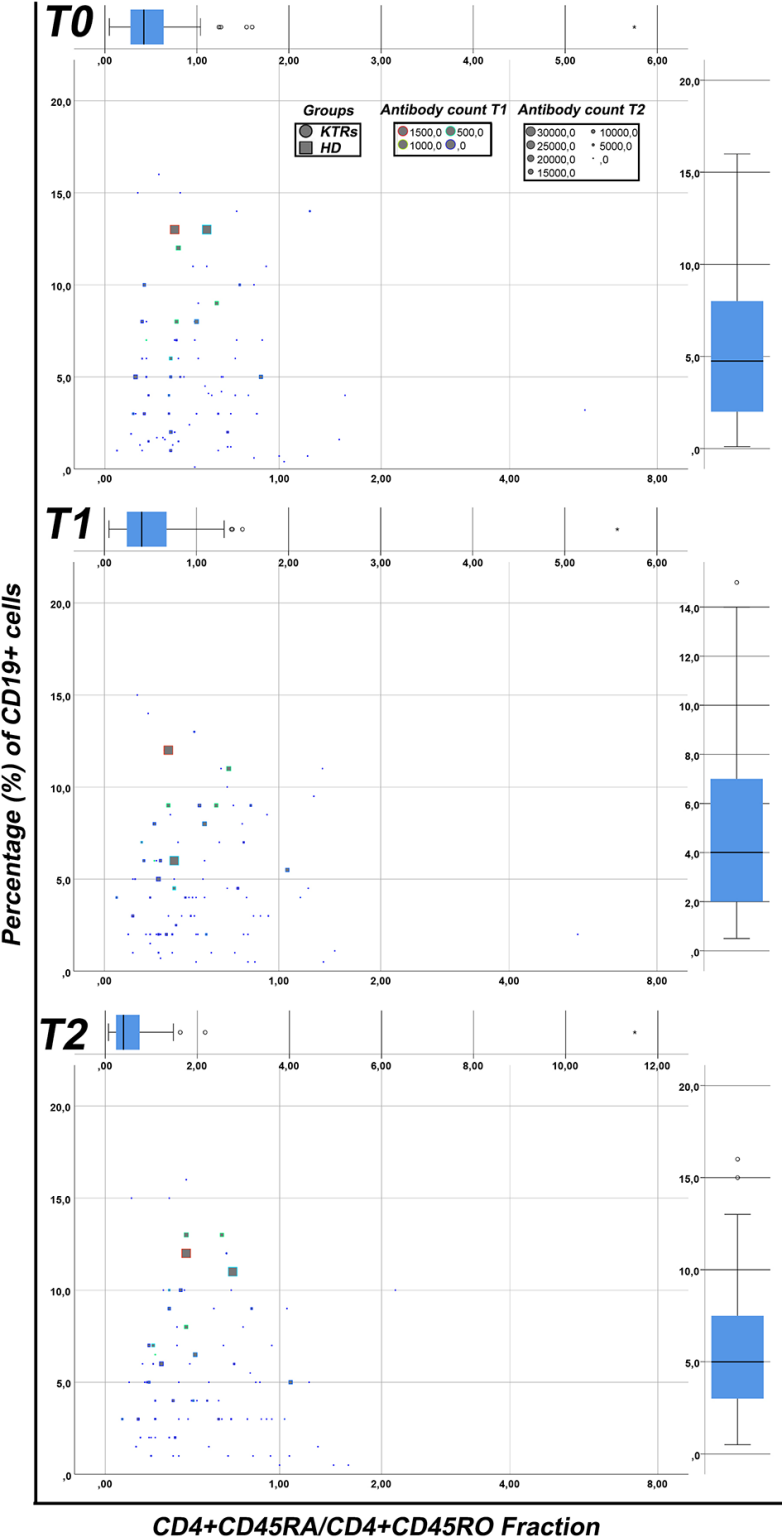
Correlation between the humoral immune response with different lymphocyte subsets. The fraction of CD4+CD45RA/CD4+CD45RO (X-axis) has been plotted against the percentage of CD19+ cells (Y-axis) in kidney transplant recipients (circular points) and hemodialysis patients (square points) patients. The antibody counts following the first (0-100 grey, 101-500 green, 501-1500 yellow, >1500 red) and the second (different sizes from small to large from 0-30000) dose of vaccine were also depicted in each point. The results are presented for T0, T1 and T2 respectively. HD, hemodialysis patients; KTRs, kidney transplant recipients; HD, hemodialysis patients; KTRs, kidney transplant recipients.

A summary of our findings regarding the correlations between the percentages of CD19+ B cells in the circulation, the fraction of CD4+CD45RO/CD4+CD45RO T helper cells and the antibody levels for HD patients and KTRs at T0, T1 and T2 is depicted in **Figure 4**. Based on a collective view of our results, even though a high variability is observed between different patients, the strongest humoral immune responses are clustered in the upper left quadrant of each diagram, which correspond to individuals with a higher percentage of CD19+ B cells as well as a larger fraction of memory CD4+ CD45RO T helper cells. HD patients tend to exhibit stronger humoral responses than KTRs. When comparing the results in the histograms corresponding to T0, T1 and T2 respectively, we conclude that the percentage of CD19+ cells did not significantly change between T0-T2. Interestingly, there were some patients who despite lower CD19+ B cell counts (depicted in the lower left quadrant), succeed in generating anti- SARS-CoV2 antibodies. In conclusion, a strong correlation appears to exist between antibody-producing CD19+ B cells, memory CD4+ T cells and anti SARS-CoV2 antibody counts.

## Discussion

The results of our study regarding the immunogenicity of the BNT162b2 vaccine in terms of eliciting an antibody response in maintenance HD patients and KTRs are in accordance with the until now available evidence regarding immunization rates in these vulnerable populations (10–12, 22–28). Notably, the findings of our study suggest that evaluation of both CD19+ lymphocytes and CD4+CD45RO helper T cell subsets in the peripheral circulation might serve as a means for estimation of the subsequent immune responses to vaccination in these patients. In particular, our findings regarding the induction of CD4+CD45RO memory T helper cells in HD patients and KTRs following vaccination is an indicator, albeit indirect, which allows us to speculate that administration of the BNT162b2 vaccine elicits a cellular immune memory response in addition antibody production.

With regard to antibody response in our patients undergoing maintenance dialysis, our findings are consistent with published data from other centers reporting that although less than one-third of HD patients develop antibodies after the first dose of BNT162b2 COVID-19 mRNA vaccine, still the overall seropositivity rate exceeds 80% after 2 doses of the BNT162b2 mRNA vaccine (22, 23). Likewise, the low seropositivity rates in our KTRs, further confirm the results from other centers reporting a weak antibody response to SARS-COV-2 vaccination with immunization rates varying from 20-50% (24–26). Furthermore, the mean levels of antibody titers detected in our HD patients who achieved a humoral immune response to vaccination, are in line with other reports, confirming substantially lower antibody titers in HD patients compared to the general population (27). Yet, in order to correctly interpret these data, one should take into account that evidence from the available studies is heterogenous in terms that antibody assays from different manufacturers were utilized, most but not all studies examined the immune response to the BNT162b2 COVID-19 mRNA vaccine whereas a few of these studies included dialysis patients with previous COVID 19 infection as well.

The classical immunosuppressive drug regimen of kidney transplantation consists of the combination of three classes of drugs, i.e. corticosteroids, a calcineurin inhibitor and an antimetabolite, which leads to improved graft survival rates yet in the setting of simultaneous profound immunosuppression. In specific, available data from research studies regarding successful implementation of vaccination strategies in organ transplantation, suggest that glucocorticoids alone dot not seem to affect vaccine efficacy (4). On the other hand, CNIs directly suppress the early antigen-dependent T-cell activation whereas the antimetabolite mycophenolate mofetil (MMF) inhibits antibody production in a T-cell independent manner, with relative sparing of T-cell responses, thus potentially affecting vaccine efficacy (4, 29–31). In specific, evidence from previous studies of influenza vaccine immunogenicity in transplant recipients indicates that MMF reduced the interleukin-4+ CD4+ T-cell frequencies as well as inhibited HLA-DR expression on B-cells together with B-cell activation, as a result decreasing production of immunoglobulin G (31, 32). In line with the above, the utilization of an intensive immunosuppressive regimen including MMF or mycophenolic acid (MPA) in our KTRs was associated with an attenuated antibody response status following vaccination against SARS COV-2.

A noteworthy observation of our study is kidney graft function was significantly related to the antibody response status in KTRs. Until now, there are scarce and controversial data in the literature regarding a possible relationship between the GFR and seroconversion rates following vaccination in patients with chronic kidney disease (33–35).

Although the evaluation of the humoral immune responses following vaccination is rather accessible and convenient, the assessment of the cellular immune responses is crucial, especially in immunosuppressed individuals such as HD patients and KTRs. Earlier studies have shown a significant reduction of total B-cell counts in the peripheral blood both in ESKD patients and KTRs (8, 36). In addition, CD19+ B-cell lymphopenia has recently been suggested as an independent predictor of all-cause and cardiovascular mortality in HD patients (8, 36). Thus, in line with previous evidence, both HD patients and KTRs in our study displayed lower levels than normal of CD19+ B lymphocytes in the circulation. A notable finding of our study is that the percentage of CD19+ B cells in the peripheral circulation is directly associated to higher antibody production following vaccination against SARS-COV2. Considering that both groups exhibit lower than normal baseline CD19+ B cell levels, our results suggest that even in such conditions of immunosuppression, there is a possibility of achieving efficient humoral responses that might ultimately offer protection against severe COVID-19 disease. Likewise, a pilot-study regarding SARS COV-2 vaccination in individuals receiving B-cell depleting treatment with rituximab, which showed that generation of an adequate antibody response coincided with the detection of peripheral CD19+ B cells (37).

Impaired renal function has been directly associated with depletion of naïve T cells, including naïve CD4+ and naïve CD8+ T lymphocytes subsets due to increased apoptosis of these cells (5, 38). Moreover, it has been shown that HD patients mount a defective effector memory CD4+ T helper cell response following vaccination with hepatitis B surface antigen (39). With regard to organ transplantation, the status of memory T cells is currently a subject of ongoing research, including their role in protective immunity as well as potential implications in allograft rejection (40). Available data regarding T cell responses following administration of the influenza vaccine in transplant recipients indicates that vaccination elicited CD4+ and CD8^+^ T-cell responses at a similar level compared to influenza infection whereas humoral immunity had a significant correlation with a CD4+ response. In addition, it appears that memory T cells may be less susceptible to the effects of conventional immunosuppression and booster immunizations are generally beneficial in these transplant recipients (41–43).

We found that the fraction of CD4+CD45RA+/CD4+CD45RO+ to be negatively associated with antibody production, thus confirming the concordance between cellular and humoral immunityand hinting that concomitant production of specialized memory immune cells against the SARS-COV2 antigens occurs together with the generation of the humoral immune response following vaccination. A similar immune response pattern has been previously observed in patients who recovered from mild-to-moderate COVID-19 disease (44). Such assumption requires further validation with measurement and characterization of specific memory immune cells with a SARS-COV2 antigen specific assay.,

Interestingly, the ratio of CD4+ to CD8+ T-helper cells progressively increased following vaccination in both hemodialysis patients and KTRs, which should be ascribed to an increasing trend of CD4+ T helper cells counts together with a simultaneous reduction of CD8+ T cells following the second vaccine dose. On the other hand, the SARS-CoV-2 infection itself has been associated with the depletion of both CD4+ and CD8+^+^ T cells counts (45, 46). Available evidence suggests that an inverted CD4+ to CD8+ T –cell ratio may indicate an impaired ability to respond to repeated antigenic stimulation in the setting of influenza vaccination (47). An inverted CD4+ to CD8+ T-cell ratio is a common finding in HD patients and if present before transplantation, it may be a risk factor for post-transplant infections (48, 49).

With regard to the other cell sub-populations, we found lower NKT cell counts in HD patients compared to KTRs following the second vaccine dose, whereas NKT cell counts increased in both patient groups following administration of the first and the second vaccine dose. Available evidence indicates that patients with ESKD display depleted invariant NKT cells, which however return to normal levels within one year following kidney transplantation (50). It should be noted that low levels of NKT cells in peripheral blood of COVID-19 subjects have been associated with the severity of the disease in these patients (51).

A remarkable finding of our study is the variability in the immune responses between immunosuppressed individuals following vaccination, a known issue concerning the general population as well (52). This variability may be attributed, among others, to the genetic background, age and the specific underlying patterns of immune-compromise, as occur in ESKD and organ transplantation. Such variable and “patient-specific” responses require sensitive techniques for the follow-up of the immune response in the setting of vaccination.

The Covid-19 pandemic disclosed the current lack of knowledge of the efficacy of vaccination strategies in the immunosuppressed individuals. There is evidence that administering a third vaccine dose in immunosuppressed populations might further enhance their immunogenicity (53). Follow-up data regarding immune responses in the setting of supplemental SARS-COV-2 vaccine doses in our patient cohorts following approval by national committees shall be made available in the future.

In conclusion, taking into account the clinical outcome of severe COVID-19, the monitoring of vaccination success in high-risk individuals such as HD patients and KTRs is of paramount importance (54). Herein, we provided a general scheme of the background immune profile of the vulnerable populations of HD patients and KTRs as well as highlighted specific traits of the humoral and cellular immune alterations following SARS COV -2 vaccination with an mRNA vaccine, thus aiming to improve the understanding of both the ability and defects of the suppressed immune system to respond to vaccination. In addition, we brought to the forefront the variation in individual immune responses which characterizes these two vulnerable patient cohorts. Thus, our methodology provides a conceptual framework for the analysis of humoral responses following BNT162b2 vaccination, based on antibody counts and flow cytometric analysis of major lymphocyte sub-populations, for monitoring vaccination success in high-risk individuals. Despite the challenges lying ahead, future research studies will further elucidate the intricate mechanisms linking background immune phenotypes to vaccine responsiveness in order to identify appropriate markers of immunogenicity and efficacy in immunosuppressed patients.

## Data Availability Statement

The original contributions presented in the study are included in the article/**Supplementary Material**. Further inquiries can be directed to the corresponding author.

## Ethics Statement

The studies involving human participants were reviewed and approved by Ethical Committee of the University Hospital of Ioannina. The patients/participants provided their written informed consent to participate in this study.

## Author Contributions

Individual contribution of each co-author is as follows: AD, design of the study, analysis and interpretation of data, drafting the article. GM, conducted flow cytometry analyses, analysis and interpretation of data, drafting and revising the article. IM, recruitment of patients, patients’ data recorded and entry, drafting the article. HP, conception and design of the study, patients’ recruitment. EP, recruitment of patients, patients’ data recorded and entry. VK, recruitment of patients, patients’ data recorded and entry. ET, recruitment of patients, patients’ data recorded and entry. GB, conducted flow cytometry analyses, analysis and interpretation of data. SP, handling and preparing patients’ blood samples, data entry. KG, conducted anti-SARS-CoV2 antibody measurements. MM, conception of the study. ED, conception and design of the study, interpretation of data, drafting and revising the article. All authors contributed to the article and approved the submitted version.

## Conflict of Interest

The authors declare that the research was conducted in the absence of any commercial or financial relationships that could be construed as a potential conflict of interest.

## Publisher’s Note

All claims expressed in this article are solely those of the authors and do not necessarily represent those of their affiliated organizations, or those of the publisher, the editors and the reviewers. Any product that may be evaluated in this article, or claim that may be made by its manufacturer, is not guaranteed or endorsed by the publisher.
