# Supplementary material for: The Humoral Immune Response to BNT162b2 Vaccine Is Associated With Circulating CD19+ B Lymphocytes and the Naïve CD45RA to Memory CD45RO CD4+ T Helper Cells Ratio in Hemodialysis Patients and Kidney Transplant Recipients
Source: Front Immunol. 2021 Dec 3;12:760249. doi: 10.3389/fimmu.2021.760249 (PMC8678464; doi:10.3389/fimmu.2021.760249)
Supplement: Supplementary file 1 [file Table_1.docx]

| **Supplementary Table 1.** Correlations between antibody response and CD19+ B cells subsets. Significant correlations are highlighted in bold letters. AB, antibody. | | | | | | | |
| --- | --- | --- | --- | --- | --- | --- | --- |
|  | | | **AB T1** | **AB T2** | **CD19+ T0** | **CD19+ T1** | **CD19+ T2** |
| Spearman's rho | **AB T1** | Correlation Coefficient | 1,000 | **,841^**^** | **,290^**^** | **,275^*^**^*^ | **,344^**^** |
|  |  | Sig. (2-tailed) | . | ,000 | ,006 | ,010 | ,001 |
|  |  | N | 88 | 88 | 88 | 88 | 88 |
|  | **AB T2** | Correlation Coefficient | **,841^**^** | 1,000 | ,204 | **,249^*^** | **,308^**^** |
|  |  | Sig. (2-tailed) | ,000 | . | ,056 | ,019 | ,004 |
|  |  | N | 88 | 88 | 88 | 88 | 88 |
|  | **CD19+ T0** | Correlation Coefficient | **,290^**^** | ,204 | 1,000 | **,860^**^** | **,885^**^** |
|  |  | Sig. (2-tailed) | ,006 | ,056 | . | ,000 | ,000 |
|  |  | N | 88 | 88 | 88 | 88 | 88 |
|  | **CD19+ T1** | Correlation Coefficient | **,275^**^** | **,249^*^** | **,860^**^** | 1,000 | **,928^**^** |
|  |  | Sig. (2-tailed) | ,010 | ,019 | ,000 | . | ,000 |
|  |  | N | 88 | 88 | 88 | 88 | 88 |
|  | **CD19+ T2** | Correlation Coefficient | **,344^**^** | **,308^**^** | **,885^**^** | **,928^**^** | 1,000 |
|  |  | Sig. (2-tailed) | ,001 | ,004 | ,000 | ,000 | . |
|  |  | N | 88 | 88 | 88 | 88 | 88 |
| **. Correlation is significant at the 0.01 level (2-tailed). | | | | | | | |
| *. Correlation is significant at the 0.05 level (2-tailed). | | | | | | | |

| **Supplementary Table 2.** Correlations between antibody response and CD4+RA/CD4+RO T helper cells cell ratio. Significant correlations are highlighted in bold letters. AB, antibody. | | | | | | | |
| --- | --- | --- | --- | --- | --- | --- | --- |
|  | | | **AB T1** | **AB T2** | **CD4+RARO T0** | **CD4+RARO T1** | **CD4+RARO T2** |
| **Spearman's rho** | **AB T1** | Correlation Coefficient | 1,000 | **,841^**^** | **-,302^**^** | **-,288^**^** | **-,317^**^** |
|  |  | Sig. (2-tailed) | . | ,000 | ,004 | ,006 | ,003 |
|  |  | N | 88 | 88 | 88 | 88 | 88 |
|  | **AB T2** | Correlation Coefficient | **,841^**^** | 1,000 | **-,311^**^** | **-,266^*^** | **-,254^*^** |
|  |  | Sig. (2-tailed) | ,000 | . | ,003 | ,012 | ,017 |
|  |  | N | 88 | 88 | 88 | 88 | 88 |
|  | **CD4+RA/CD4+RO**  **T0** | Correlation Coefficient | **-,302^**^** | **-,311^**^** | 1,000 | **,748^**^** | **,846^**^** |
|  |  | Sig. (2-tailed) | ,004 | ,003 | . | ,000 | ,000 |
|  |  | N | 88 | 88 | 88 | 88 | 88 |
|  | **CD4+RA/CD4+RO**  **T1** | Correlation Coefficient | **-,288^**^** | **-,266^*^** | **,748^**^** | 1,000 | **,771^**^** |
|  |  | Sig. (2-tailed) | ,006 | ,012 | ,000 | . | ,000 |
|  |  | N | 88 | 88 | 88 | 88 | 88 |
|  | **CD4+RA/CD4+RO**  **T2** | Correlation Coefficient | **-,317^**^** | **-,254^*^** | **,846^**^** | **,771^**^** | 1,000 |
|  |  | Sig. (2-tailed) | ,003 | ,017 | ,000 | ,000 | . |
|  |  | N | 88 | 88 | 88 | 88 | 88 |
| **. Correlation is significant at the 0.01 level (2-tailed). | | | | | | | |
| *. Correlation is significant at the 0.05 level (2-tailed). | | | | | | | |
